# Amygdala volume changes as a potential marker of multiple sclerosis progression: links to EDSS scores and PIRA

**DOI:** 10.3389/fimmu.2025.1640607

**Published:** 2025-08-26

**Authors:** Aleksandra Pogoda-Wesołowska, Ignacy Stachura, Nina Sługocka, Marta Kania-Pudło, Jacek Staszewski, Adam Stępień

**Affiliations:** ^1^ Neurology Clinic, Military Institute of Medicine – National Research Institute, Warsaw, Poland; ^2^ Faculty of Physics, University of Warsaw, Warsaw, Poland; ^3^ Faculty of Medicine, University of Warsaw, Warsaw, Poland; ^4^ Department of Medical Radiology, Military Institute of Medicine – National Research Institute, Warsaw, Poland

**Keywords:** multiple sclerosis, disability progression, EDSS, PIRA, atrophy, amygdala

## Abstract

**Introduction:**

Brain atrophy may be a promising marker of relapsing-remitting multiple sclerosis (RRMS) progression, yet it remains underutilized in clinical practice. This exploratory study evaluated correlations between disability—as measured by the Expanded Disability Status Scale (EDSS) and progression independent of relapse activity (PIRA)—and volumetric changes in RRMS patients treated with cladribine tablets (CLAD) or alemtuzumab (ALEM).

**Methods:**

Clinical and magnetic resonance imaging (MRI) data from patients with RRMS were retrospectively analyzed at four time points: pretreatment and annually over three years of follow-up. Volumetric measurements were obtained using FreeSurfer. Annual volumetric and EDSS changes were pooled together to assess short-term associations and patient-wise longitudinal analyses were performed.

**Results:**

33 patients treated with CLAD and 19 patients treated with ALEM were included. Analyzing year-to-year correlations, a significant positive correlation was found between EDSS and amygdala volume changes (p = 0.00009, η²=0,15657). It was also observed for the pallidum (p=0,02605, η²=0,05384). On the contrary, a negative correlation between thalamic volume changes and EDSS in CLAD group was noted (p=0,04551, η²=0,07203).When comparing annual percentage volume changes across three groups—years with EDSS progression (n = 10), regression (n = 11), and no changes (n = 74)—significant differences were reported in amygdala (p=0,00640; 1.98%, -4%, -0.8%), thalamus (p = 0,04390; -0.54%, 2.98%, 0.1%) and pallidum (p = 0,02904; 1.98%, -6.96%, -0.23%). Finally, among the 10 patients with EDSS progression, an increase in amygdala volume was observed in 3 patients with PIRA, whereas it was not seen in the 7 patients whose EDSS progression was associated with relapsing activity (p = 0.0188; 4.60% vs. 0.004%).

**Conclusion:**

Over three years of follow-up in RRMS patients, EDSS progression was positively associated with increases in amygdala—and, to a lesser extent, pallidum—volumes, while worsening disability correlated with thalamic atrophy. Notably, amygdala enlargement was exclusive to patients with PIRA versus relapse-associated worsening, highlighting its potential as a volumetric biomarker of disease progression. However it was exploratory, hypothesis-generating observation and further studies are warranted to validate these findings and elucidate the underlying mechanisms.

## 1 Introduction

Multiple sclerosis (MS) is a chronic, autoimmune disease that affects the central nervous system (CNS) and is typified by neuroinflammation, axonal damage, and progressive demyelination (1). Neurodegeneration was previously considered a late-stage phenomenon with limited clinical significance. However, it is now recognized that it is associated with acute inflammation from the early stages of MS and is a major factor in irreversible disability (2). MS is now considered to be a simultaneous disease with two components, in which the relative contribution of different pathological disease processes (inflammation and neurodegeneration) to the development of disability, their relationship, and their evolution over time coexist.

Previous studies have focused mainly on inflammatory processes in MS; therefore, knowledge of neurodegeneration is still limited, especially with respect to the effect of pharmacological interventions on the extent and pattern of brain atrophy and its correlation with clinical progression. Despite significant progress in the clinical treatment of MS patients, the mechanisms driving disability accumulation are not fully understood (3). It is widely believed that disability accumulation may result from neuroinflammatory events occurring in clinical relapses (relapse-associated worsening - RAW). However, an increasing number of articles discuss progression independent of relapse activity (PIRA). PIRA reflects underlying neurodegeneration that is not associated with acute inflammatory relapses and is now recognized as a key contributor to sustained disability across the MS spectrum, including early relapsing-remitting stages (4). Interestingly, while increasing disability is characteristic of progressive MS courses, PIRA has recently emerged as a key clinical feature also in relapsing MS (RMS) (5). Understanding PIRA may lead to better targeted therapies that address the underlying neurodegenerative processes driving disability progression. PIRA may therefore be considered as a potential clinical trial outcome to measure the efficacy of therapy in preventing or slowing the progression of disability, even in the absence of relapses. The pathophysiological determinants of PIRA remain elusive, although it is likely that PIRA is associated with increased diffuse neuroaxonal loss. The Expanded Disability Status Scale (EDSS) remains the most widely used clinical tool to evaluate disability in MS, yet it does not fully capture the subtleties of neurodegenerative progression, especially when dissociated from relapse activity (6).

A growing body of research highlights the potential of quantitative magnetic resonance imaging (MRI)-based markers—particularly brain volume loss—as indicators of neurodegeneration and disease progression. Assessment of brain atrophy by MRI provides *in vivo* quantification of ongoing neurodegenerative processes (5). Brain tissue loss may result from acute focal neuroinflammatory events as well as more diffuse primary or secondary neurodegenerative processes that occur independently of lesion activity. According to collected evidence, MS pathology may affect both white (WM) and grey matter (GM), and neurodegenerative changes are a significant contributor to long-term disability. Brain atrophy, a reflection of cumulative tissue loss, is increasingly recognized as a critical aspect in assessing the burden of MS and is becoming a sensitive indication of the disease’s progression (4). However, there is still limited routine use of volumetric MRI measurements to track the development of disease, despite their clinical importance. The extent to which volumetric changes in specific brain regions correlate with clinical disability as measured by the EDSS and PIRA remains insufficiently defined, especially in patients undergoing highly effective therapies.

This study aimed to evaluate the clinical relevance of brain atrophy by examining its associations with EDSS progression and PIRA in RRMS patients treated with cladribine tablets (CLAD) or alemtuzumab (ALEM). Regional atrophy patterns between patients with PIRA and those with RAW were also compared. Longitudinal MRI data were used to quantify brain structure volume changes and their relationship to disability evolution. Our findings may enhance understanding of brain atrophy as a biomarker of MS progression and its utility for monitoring treatment efficacy.

## 2 Methods

### 2.1 Participants

A retrospective, observational, longitudinal study was conducted at the Neurology Clinic of the Military Institute of Medicine – National Research Institute (MIM-NRI). Patients with RRMS diagnosed according to the 2017 McDonald criteria, treated with immune reconstitution therapies (IRT)— CLAD and ALEM were recruited to the study. Demographic data (age, gender, disease duration, comorbidities, number of previous therapies, adverse events, reason for treatment change) and clinical data (annualized relapse rate (ARR), EDSS score, number of new lesions on T2-weighted (T2) magnetic resonance imaging (MRI) including contrast-enhancing (Gd+) lesions) were collected, as well as MRI examinations at 4 time points: before treatment, 1 year after treatment, 2 years after treatment, and 3 years after treatment.

Confirmed disability progression was defined as a minimum increase in the EDSS of 1.5, 1.0, or 0.5 from a baseline level of 0, 1.0–5.0, and 5.5, respectively. PIRA was defined as an episode of confirmed disability progression with no relapse during the 90 days before the EDSS increase and during the 6-month period between the EDSS increase and the confirmation of disability progression.

All clinical, demographic and MRI data were fully anonymized prior to analysis; patient identifiers, including ID and PESEL numbers, were removed and replaced by study-specific acronyms. As this was a retrospective review of existing data, individual written informed consent was not required. The study protocol was submitted to the MIM-NRI Bioethics Committee, which issued Resolution No. 54/24 on October 16, 2024, confirming that the project did not require further committee review.

### 2.2 MRI examination

All patients were scanned using the same MRI operating system on a General Electric Discovery MR750W3T 3.0 Tesla in the MIM-NRI Magnetic Resonance Imaging Laboratory. A standard protocol for MS was used, including sagittalis (sag) and axialis (ax) T2-weighted gradient-echo (T2) PROPELLER sequence, ax fluid-attenuated inversion recovery sequence (FLAIR), sag CUBE FLAIR sequence, ax diffusion-weighted imaging (DWI) sequence, three- dimensional (3D) susceptibility-weighted angiography (SWAN) sequence and ax 3D T1 pre- and post-contrast sequences. Baseline MRI examinations were performed before the initiation of therapy. As part of routine follow-up of new T2 lesions (including Gd+ lesions), yearly control MRI examinations were performed. MRI data were acquired from the hospital Alteris system as DICOM file folders in 0.6 mm axial 3D T1 and anonymized.

### 2.3 Volumetric analysis

MRI examinations were analyzed volumetrically using Freesurfer software (version 7.4.0). Segmentation of brain structures based on each subject 3D T1-weighted MRI was performed automatically for each patient using automated longitudinal FreeSurfer processing pipeline (7, 8). No manual editing was performed to keep methods as automated as possible, and scans with segmentation errors/failures were excluded. Volumetric measurements were also manually verified by two qualified neurologists. MRI examinations of poor quality (unable to analyze by Freesurfer software due to, for example, artifacts, different MRI protocol), performed after a switch to therapy different than CLAD or ALEM, and performed less than 8 weeks after intravenous steroid administration were excluded from the analysis. Assessment of the progression of brain atrophy was based on the comparison of volumes of different structures at different timepoints of the treatment period.

### 2.4 Statistical analysis

All regional volumes were extracted from the FreeSurfer longitudinal pipeline and normalized to each subject’s estimated total intracranial volume (eTIV)—which remains constant across all timepoints—yielding unitless percentages of eTIV. For every ROI and EDSS score, absolute change was computed between corresponding visits.

We assessed correlations via two approaches:

1. Pooled “patient-year” analysis.

Annual volume and EDSS changes were pooled across all subjects and years (n = 97 patient-years). This approach, we believe, encompasses short-term associations between EDSS and volumetric changes. The relationship between annual volumetric changes and annual EDSS changes was assessed using a multiple linear regression model, with age, sex, baseline EDSS, and number of prior therapies included as covariates. Residuals were assessed for normality using histograms, and no significant deviations were observed. Adjusted p-values and effect sizes (η²) were reported for EDSS change only, as no significant associations were found for the covariates.

2. Within-subject longitudinal analysis.

In a subset of patients with longer-term follow-up, volumetric changes over two-year (n = 42) and three-year (n = 16) intervals were regressed against corresponding EDSS changes during the same periods. Due to limited sample sizes, simple linear regression was used. Pearson correlation coefficients and two-sided p-values were reported.

To compare structural atrophy patterns across different disability trajectories—years marked by EDSS progression, regression, or stability—we calculated the percentage change in volume (PCV) as (volume_follow-up − volume_baseline)/volume_baseline × 100%. Differences among the three groups were tested using ANCOVA-like linear model with age, sex, baseline EDSS, and number of prior therapies used as covariates. In all cases, Levene’s test indicated homogeneity of variances across groups (p > 0.05). The normality of residuals was assessed visually using histograms, and no significant deviations were detected. Adjusted p-values and effect sizes (η²) are reported for the EDSS trajectory groups only, since no significant associations were observed for the covariates. For this comparison, *post-hoc* power analysis was conducted. Sample size was estimated based on the observed effect sizes, assuming α = 0.05 and 80% power for one-way ANOVA.

In this study, separate analyses were performed for individual brain structures. Each statistical test was treated as addressing a distinct hypothesis regarding the absence of correlation between EDSS changes and volumetric changes in a specific brain region. The global (joint) hypothesis of no correlation between EDSS and any brain structure was not of interest. Therefore, following the reasoning outlined by Rubinn, Althouse and Rothman, control of the family-wise error rate was not required (9–11). Consequently, no multiple comparison corrections were applied. To ensure transparency and clarity, we reported all statistical tests along with their corresponding effect sizes and unadjusted (descriptive) p-values.

## 3 Results

### 3.1 Baseline characteristic of the groups

The study included 33 patients treated with CLAD (mean age 37.6 years; mean disease duration 8.77 years; 73% women, median baseline EDSS 3; mean baseline T2/Gd+ lesions 3.39/1.27; percentage of patients with relapses before treatment initiation- 84.8%) and 19 patients treated with ALEM (mean age 33.3 years; mean disease duration 7.19 years; 100% women, median baseline EDSS 4.0; mean baseline T2/Gd+ lesions 6.26/2.89; percentage of patients with relapses before treatment initiation- 89.5%). The initial comparison of groups was presented in **Table 1**. As can be seen, at baseline the treatment groups were similar in terms of age, duration of disease and number of previous disease modifying therapies (DMTs). In the CLAD group, most patients had previously been treated with dimethyl fumarate (55%), glatiramer acetate (9%), ocrelizumab (9%) and fingolimod (9%), similarly to the ALEM group—dimethyl fumarate 26%, fingolimod 21%, natalizumab (16%) and interferon beta-1a 16%. However, groups differed in terms of ARR and disability assessment on the EDSS scale—patients treated with ALEM were characterized by higher disease activity before the start of treatment in terms of relapses and number of T2 and had higher median baseline EDSS score.

**Table 1:** The initial comparison of the treatment groups.

	CLAD	ALEM	P-value	Cohen’s d
Age of onset	37.6	33.3	0.159	0,411028
Disease duration	8.77	7.19	0.227	0,328188
Previous DMTs	2.24	2.78	0.161	0,441635
Relapses	1.30	2.26	0.002	0,935618
EDSS score	3.05	3.97	0.034	0,626044
T2 lesions	3.39	6.26	0.010	0,702715
GD+ lesions	1.27	2.89	0.309	0,429129

DMTs, disease modifying therapies; T2 lesions, new lesions on T2-weighted magnetic resonance imaging scans; Gd+ lesions, gadolinium enhancing lesions. Values represent group means. P-values were calculated using Student’s t-test or Wilcoxon rank-sum test, as appropriate. Cohen’s d - effect size. CLAD - cladribine tablets. ALEM - alemtuzumab. The green background color was used to highlight statistically significant values.

### 3.2 Clinical changes within groups in subsequent years of treatment

In the CLAD group, the percentage of patients with relapses decreased from 84.8% before treatment to 24.2% after the first year of treatment, 12.5% after 2 years of treatment and was at the level of 46.2% after the third year of observation. The mean number of new T2 and Gd+ lesions was reduced from 3.39 and 1.27 before treatment to 0.56 and 0.22 after the first year of CLAD therapy, to 0 and 0 after 2 years of treatment and was 0.85 and 0.23 after 3 years of follow-up. The EDSS score, the median of which was 3 before treatment, remained stable after the first and second year of treatment, while after 3 years of observation it was 3.5. During the three-year follow-up, three patients experienced PIRA (one per year). In the ALEM group, the percentage of patients with relapses after 3 years of therapy was 23% and decreased from 89.5% before treatment (after the first year it was 21% and after 2 years of treatment 11.1%). The mean number of new T2 and Gd+ lesions before treatment was 6.26 and 2.89 and was reduced to 0.26 and 0.11 after 1 year of therapy, to 0.17 and 0.11 after 2 years of treatment. After 3 years it was 0.92 and 0.5. The median EDSS score before treatment was 4.0 and decreased to 3.0 after 1 year of treatment, remaining stable after 2 and 3 years of treatment. After three years of therapy, none of the patients experienced PIRA.

### 3.3 Correlation of volumetric changes with clinical progression of the disease

#### 3.3.1 Year-to-year correlations between EDSS changes and volume changes

**Figure 1:**
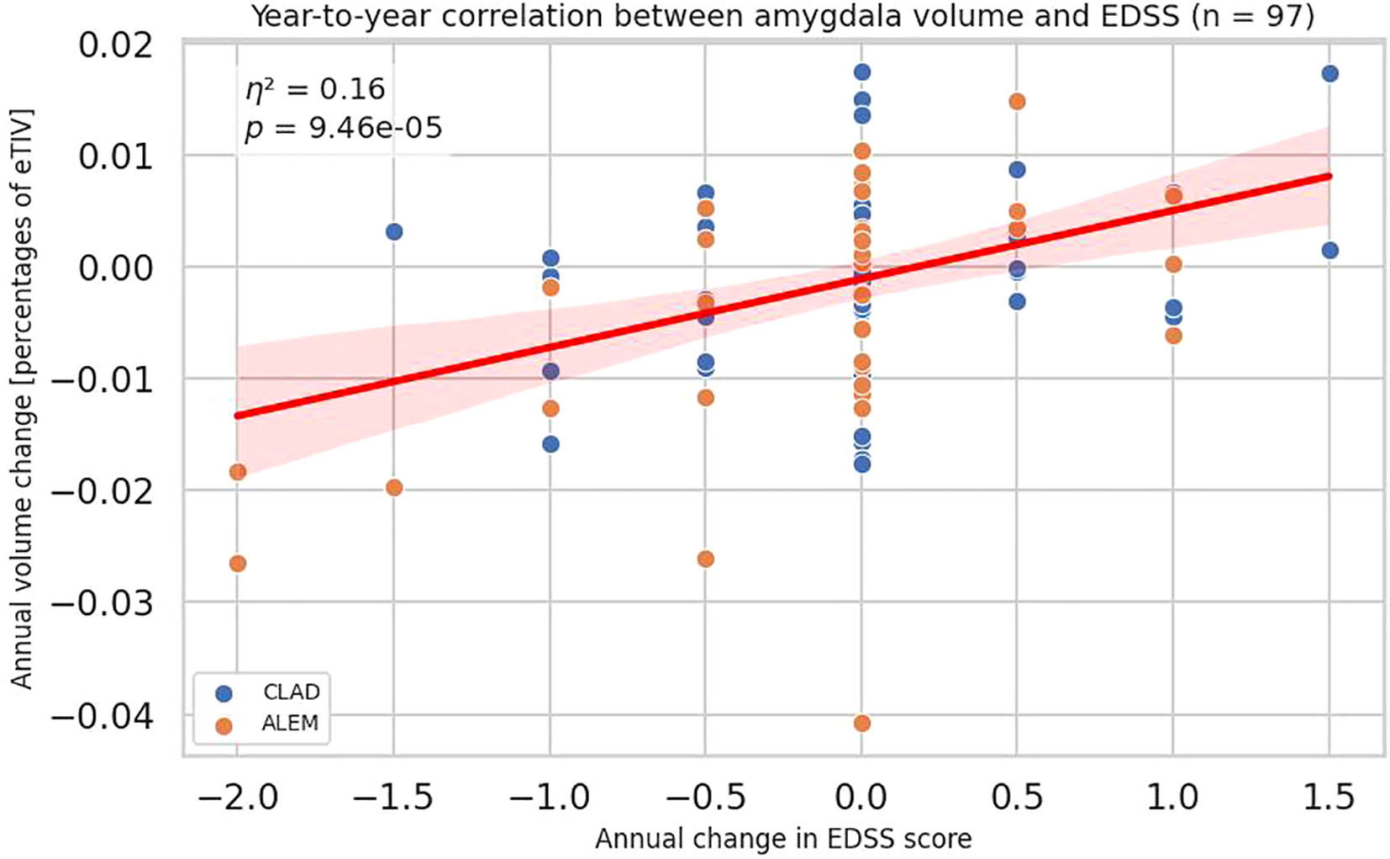
Association between annual changes in Expanded Disability Status Scale (EDSS) and corresponding annual changes in amygdala volume. Each point represents a patient-year, pooled across a 3-year period. P-value and eta-squared (η²) were obtained from a linear model and are adjusted by sex, age, baseline EDSS, and number of previous disease modifying therapies (DMTs). A linear regression line with 95% confidence interval was shown.

**Figure 2:**
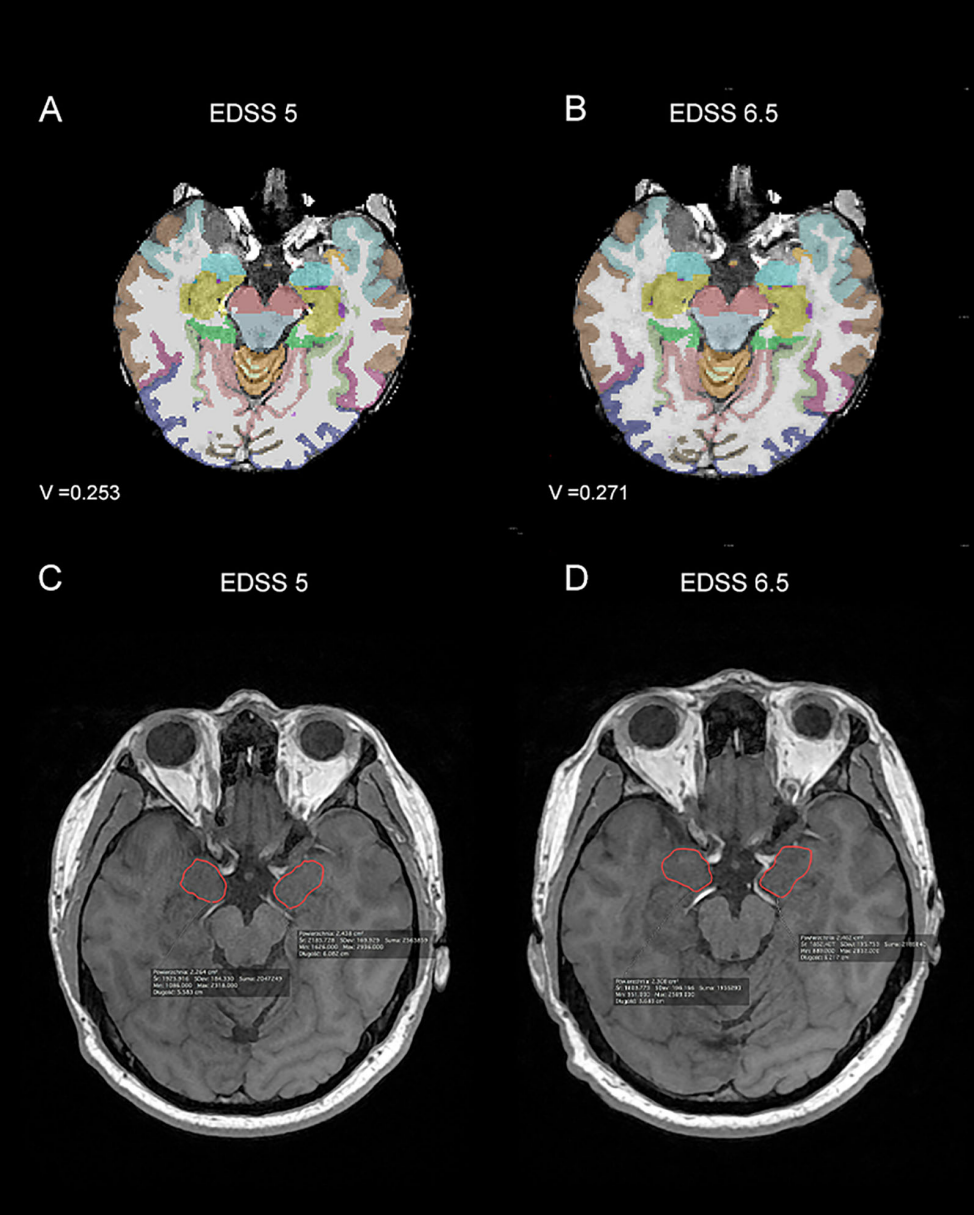
Magnetic resonance imagining (MRI) scans from patient with Expanded Disability Status Scale (EDSS) progression - at time point T0 **(A, C)** the EDSS was 5, while at time point T1 **(B, D)** it was 6.5. In parts **(A, B)** images from Freesurfer software at time points T0 - baseline **(A)** and T1 – after one year of treatment **(B)** with the amygdala volume (V) marked were shown. In parts **(C, D)** 3D T1-weighted MRI images from the Alteris system at time points T0- baseline **(C)** and T1 – after one year of treatment **(D)**, with the dimensions of the amygdala marked manually by the radiologist, were shown. V, volume of amygdala.

The obtained data indicated an increase in the volume of the amygdala in patients with progression on the EDSS scale and a decrease in its volume in patients with improvement in disability measured by the EDSS scale (all patients p=0,00009, η²=0,15657; CLAD group p=0,06972, η²=0,05963; ALEM group p=0,00310, η²=0,24923) (**Figures 1**, **2**). In the calculations, all years of follow-up were analyzed together (patient-years). This correlation had a similar strength across all three years. When examining annual correlations from each individual year, the slope coefficients, obtained from the linear regression, were 0.006, 0.0059, and 0.0055, respectively. These values suggested that the association was stable over time and not dependent on the specific treatment year. This supported our decision to report the results of a pooled analysis.

**Figure 3:**
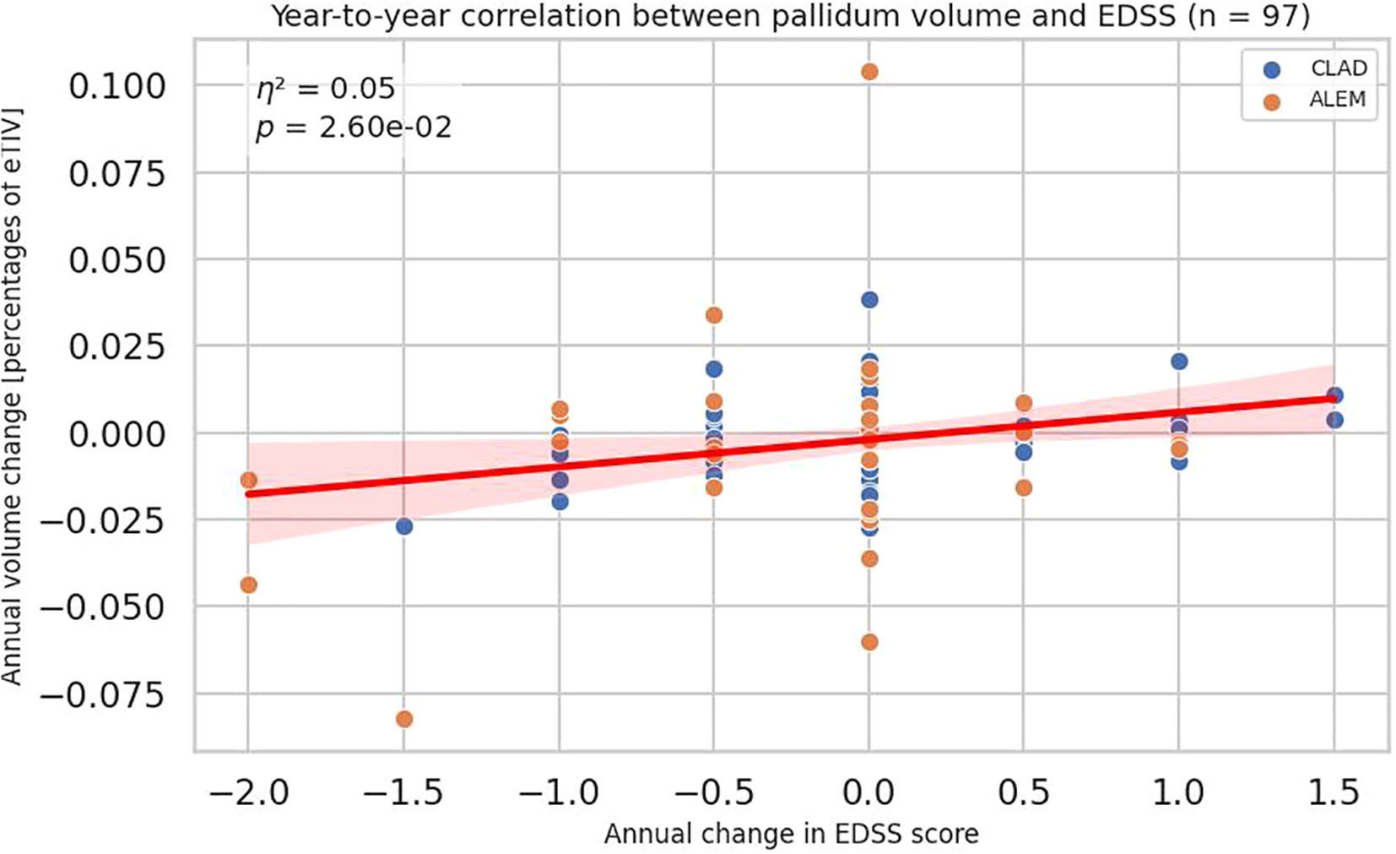
Correlation between annual changes in Expanded Disability Status Scale (EDSS) and corresponding annual changes in pallidum volume. Each point represents a patient-year, pooled across a 3-year period. P-value and eta-squared (η²) were obtained from a linear model and are adjusted by sex, age, baseline EDSS, and number of previous disease modifying therapies (DMTs). A linear regression line with 95% confidence interval is shown.

A similar, but slightly weaker, correlation is also visible for pallidum (all p-value 0,02605, η²=0,05384; CLAD p-value 0,08444, η²=0,05413; ALEM p-value 0,15180, η²=0,06511) (**Figure 3**). However, this association was not consistent across the individual years, which complicates its possible interpretation. The strongest correlation was observed for changes in the first year of observation, which may be related to the largest number of results obtained in this period, as well as the largest range of changes in the EDSS scale (in the subsequent treatment years the number of patients with EDSS change was low).

Interestingly, in the group of patients treated with CLAD, a negative correlation between thalamic volume changes and EDSS was noted (p=0.04551, η²=0.07203).

Year-to-year correlations between EDSS changes and volume changes were presented in **Supplementary Table S1**.

**Figure 4:**
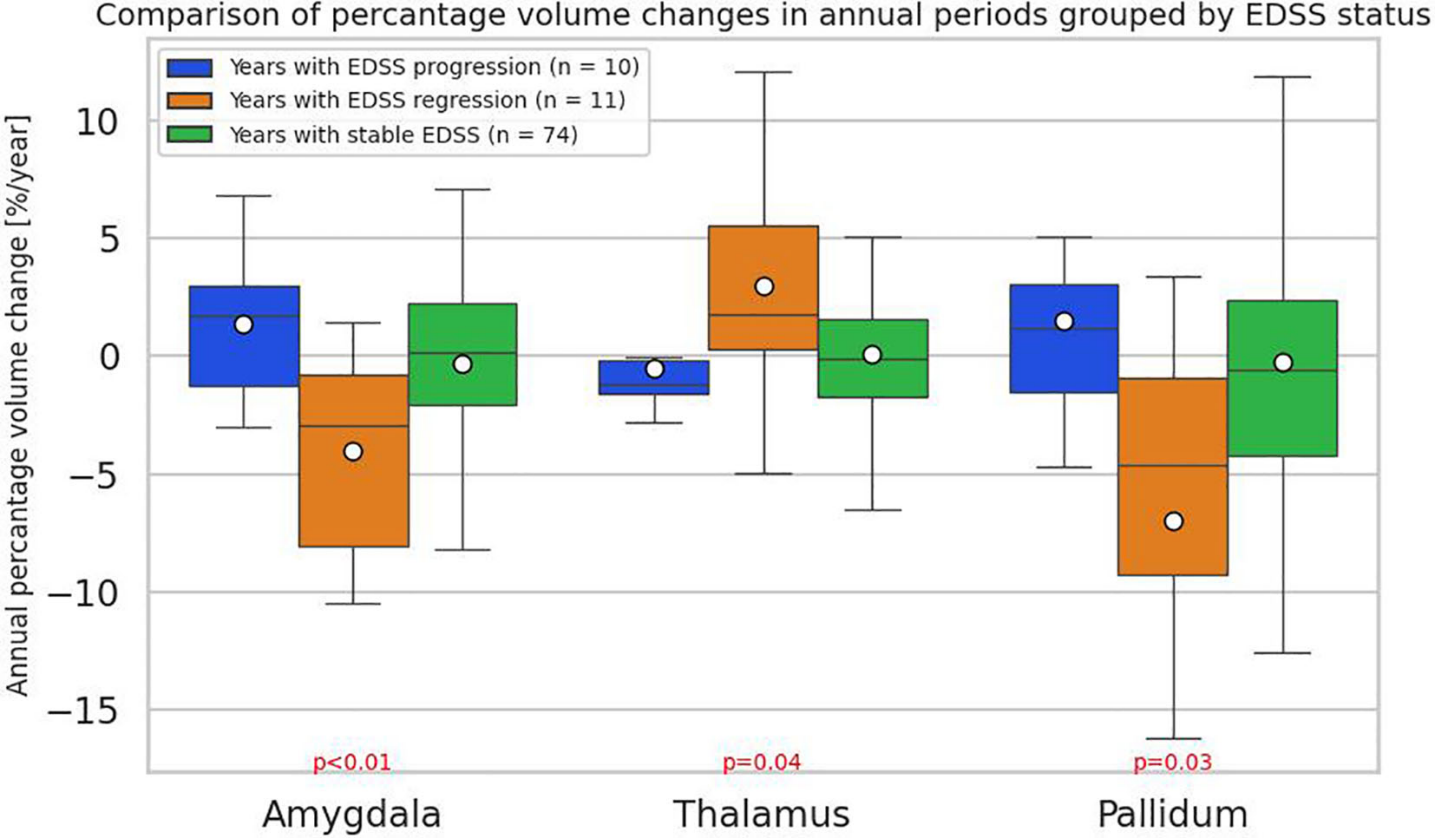
Comparison of annual percentage volume changes in the amygdala, thalamus, and pallidum, grouped by annual Expanded Disability Status Scale (EDSS) changes: regression(n = 10), progression (n = 11), and stabilization (n = 74). The division into groups with regression or progression of EDSS was defined as for progression independent of relapse activity (PIRA). The boxes represent the interquartile range (IQR) with the horizontal line indicating the median. Whiskers extend to data points within 1.5 × IQR, dots represent group means. P-values and effect sizes (eta-squared (η²)) were obtained from an ANCOVA-like linear model and are adjusted by sex, age, baseline EDSS, and number of previous disease modifying therapies (DMTs).

To further assess the relationship between the EDSS changes and volumetric changes, years were divided into three groups —years with improvement in terms of disability (n = 10), disability progression (n = 11), and the rest (stable patients) (n = 74) and ANCOVA-like model was performed. The division into groups with regression or progression of disability was defined as for PIRA. Significant differences were found in three brain regions: amygdala (p=0,00640; 1.98%, -4%, -0.8%), thalamus (p = 0,04390; -0.54%, 2.98%, 0.1%) and pallidum (p = 0,02904; 1.98%, -6.96%, -0.23%) (**Supplementary Table S2**). It was observed that an increase in EDSS was associated with an increase in the volume of the amygdala, and a decrease with a decrease in its volume. A similar situation, with lower statistical significance, was observed for the pallidum. The opposite situation was noticed for the thalamus, where an increase in volume in years with improvement in terms of disability was most apparent. As can be seen in **Figure 4**, in years with stable EDSS in all three structures, there was barely any volume change observed.

#### 3.3.2 Correlations between EDSS change and volume change over the entire three-year period and during the first two years

**Figure 5:**
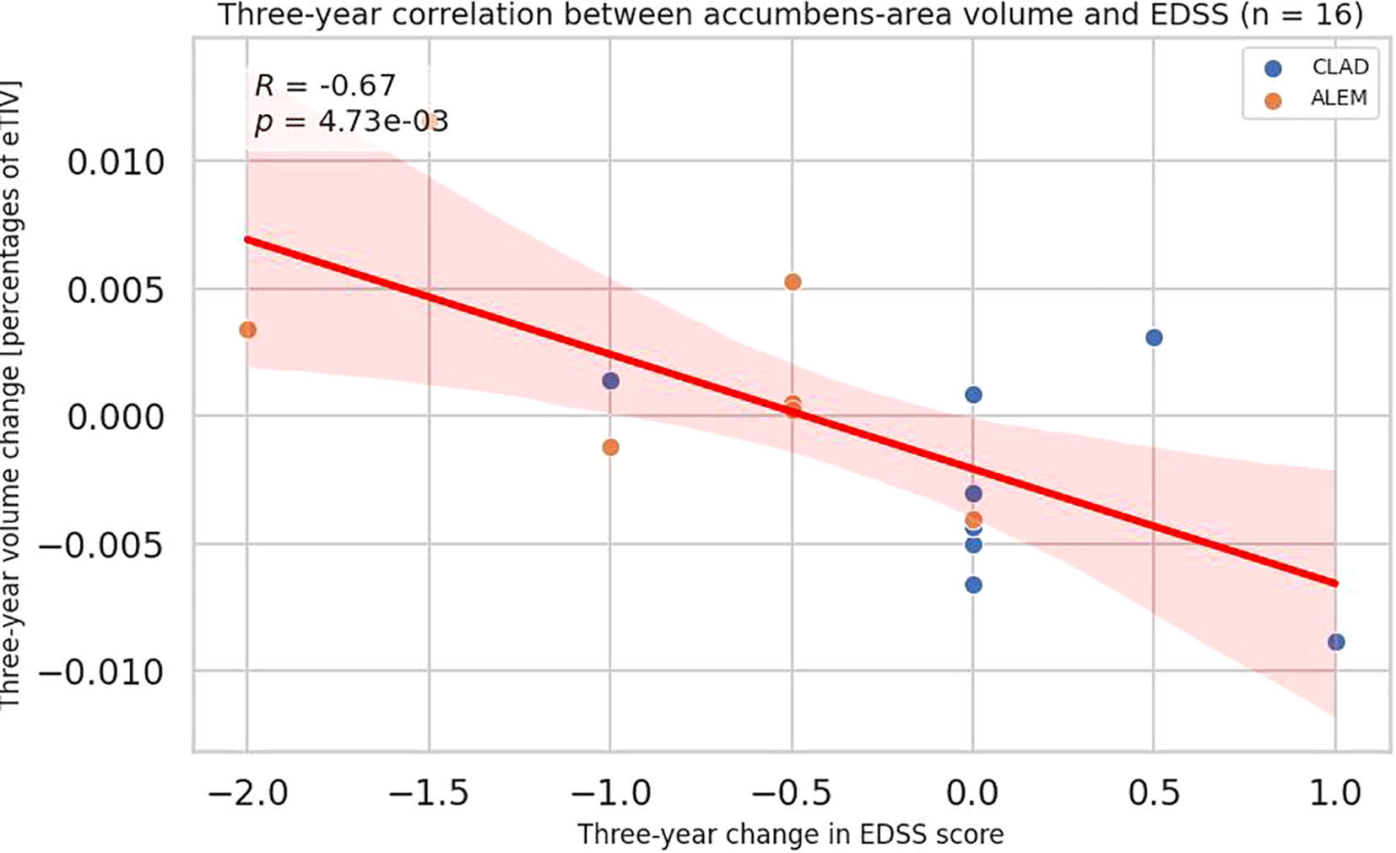
Correlation between 3-year changes in Expanded Disability Status Scale (EDSS) and corresponding changes in accumbens-area volume from the patient-wise analysis. A linear regression line with 95% confidence interval is shown. The p-value and Pearson’s correlation coefficient (R) from the linear regression model are shown.

Analyzing the correlations between three-year changes in volume and changes in the EDSS scale (n = 16), a negative correlation with the accumbens area was noted (all patients p=0.006, R=-0.67; CLAD group p=0.180; ALEM group=0.164) (**Figure 5**, **Supplementary Table S3**). For amygdala the trend was the same as it was for individual years, but the result was not significant. Perhaps it was due to the small number of patients.

**Figure 6:**
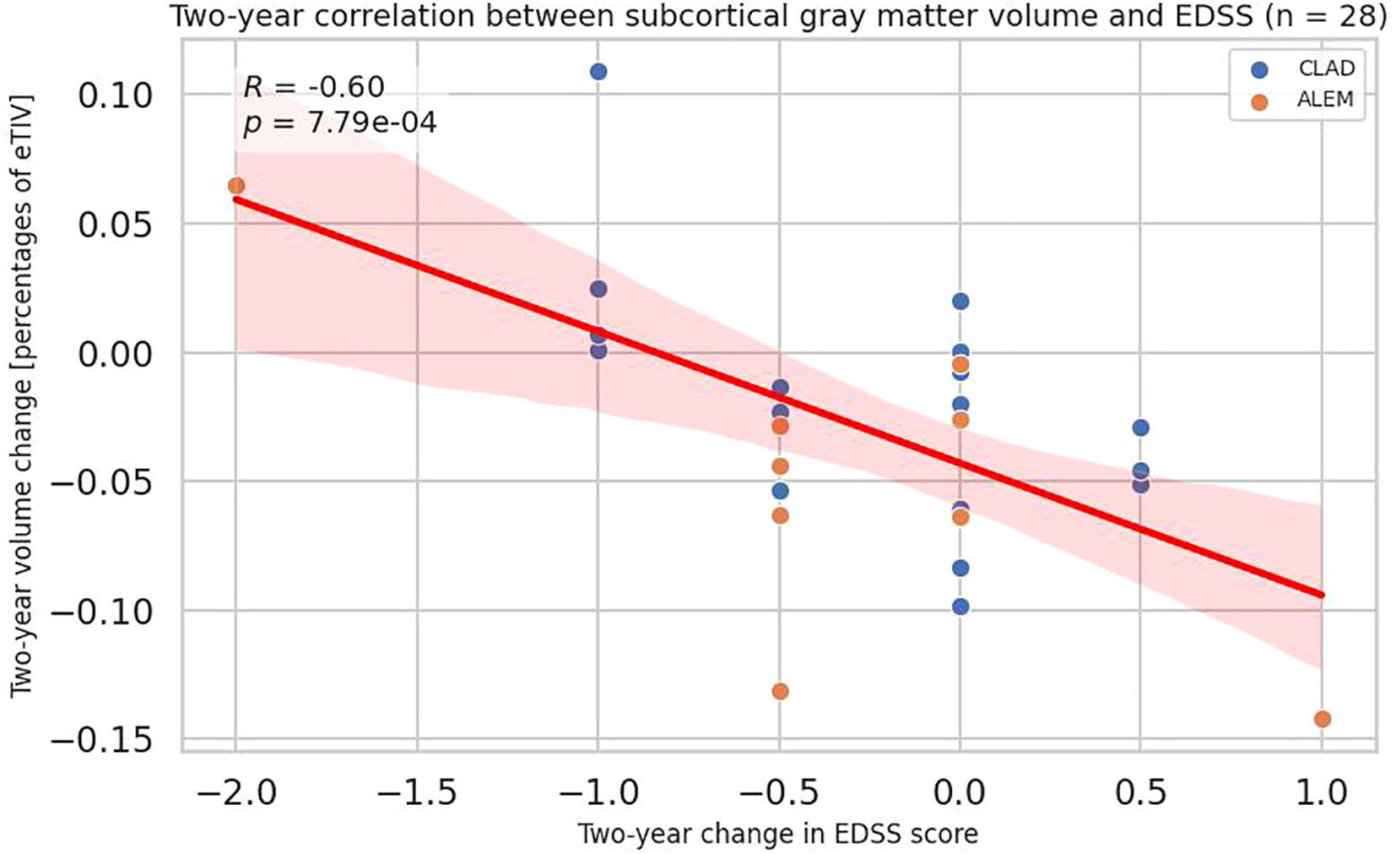
Correlation between 2-year changes in Expanded Disability Status Scale (EDSS) and corresponding changes in subcortical gray matter volume from the patient-wise analysis. A linear regression line with 95% confidence interval is shown. The p-value and Pearson’s correlation coefficient (R) from the linear regression model are shown.

Considering the first two years of observation, EDSS changes negatively correlated with the changes in subcortical gray matter volume (all patients p=0.0008, CLAD group p=0.011; ALEM group p=0.020), accumbens area (all patients p=0.046, CLAD group p=0.421, ALEM group p=0.120) and thalamus (all patients p=0.001; CLAD group p=0.109, ALEM group p=0.011) (**Figure 6**, **Supplementary Table 4**).

It should be pointed up here that the presented study was primarily exploratory in nature, and its results generate only preliminary research hypotheses that should be confirmed in further analyses on larger patient groups. To guide future research, we performed a *post hoc* analysis estimating the number of study participants in each group necessary to detect statistically significant differences, assuming the effect sizes observed in our study, using one-way analysis of variance with α = 0.05 and 80% power. The results were included in **Supplementary Table S5**.

#### 3.3.3 PIRA association with atrophy pattern

Additionally, among the 10 patients with EDSS progression, an increase in amygdala volume was observed in all three patients with PIRA, whereas this increase was not seen in the seven patients whose EDSS progression was associated with relapsing activity (p = 0.0188; 4.60% vs. 0.004%). Between time points 0 and 3 or time points 1–3, respectively 1 and 3 subjects had EDSS progression (as defined by PIRA). When specific years were indicated, there were 10 “patient-years” with EDSS progression. Of these 10 patient-years, 3 had PIRA (EDSS progression without disease activity). Time points where EDSS progression was associated with PIRA and where it was RAW were compared. The resulting groups contained small numbers of subjects (3 vs. 7), but an increase in amygdala volume in the first group compared to the second was noted (PIRA vs. EDSS progression accompanied by a clinical relapse or a new MRI lesions: p = 0.0188).

## 4 Discussion

In the present study changes in the volume of specific brain structures over time were found to be significantly associated with disease progression measured by the EDSS scale and PIRA. However, due to the limited number of patients, it should be emphasized that this observation was exploratory and its results can only constitute some kind of research hypotheses that require further studies and confirmation in larger cohorts.

First, in the analysis of year-to-year volumetric changes, an increase in the volume of the amygdala was demonstrated in patients with progression on the EDSS scale, and a decrease in its volume in patients with improvement in disability measured by the EDSS scale. A similar, but slightly weaker correlation was also seen for pallidum. Moreover, when comparing the annual percentage changes in volume in the three groups—years with EDSS progression, regression, and no change—it was also observed that EDSS changes positively correlated with amygdala volume change. This was also noted for the pallidum.

The amygdala, a key component of the limbic system, regulates emotional processing—including fear, aggression, emotional memory, and social cognition—and is implicated in neuropsychiatric disorders (such as depression and post-traumatic stress disorder) and the emotional aspects of chronic pain (12).

Several studies supported our finding that amygdala volume may be linked to clinical progression in MS. Bozhenko et al. found that larger amygdala volumes were associated with pain prioritization, anxiety, and depression in MS patients, despite global brain atrophy (13).In MS patients who considered pain to be the most distressing disease’s syndrome, the amygdala volume was larger than in MS patients who did not who did not think so (p < 0.001).

In contrast, Kever et al. reported that larger amygdala volume was related to higher social network structure, while Batista et al. showed that social cognition deficits in MS correlated with subcortical volume, with the amygdala emerging as the only predictor of performance in ToM tests (eyes test, video test) (14, 15). Furthermore, Pitteri et al. demonstrated that amygdala lesion load was a significant predictor of impairments in emotion recognition and empathy (16).

Moreover, neuroimaging studies, such as that by Meyer-Arndt et al., suggested that altered amygdala-prefrontal connectivity may underlie emotion regulation deficits in MS patients with depression (17). The clinical significance of the prefrontal cortex and amygdala as neural substrates of impaired emotion regulation has been supported by a variety of neuroimaging studies of depression (18–20).

In addition, data from Dworsky-Fried et al. also highlighted the role of central amygdala microglial activation in pain hypersensitivity in experimental autoimmune encephalomyelitis (EAE), potentially explaining altered pain processing in MS (21). Finally, Green et al. reported that reduced amygdala volume in pediatric-onset MS correlated with poorer memory and social functioning, whereas larger amygdala volumes were linked to better psychosocial outcomes (22). After controlling for whole brain volume, right amygdala volume was positively associated with visual memory; left amygdala volume was a stronger predictor of parent-reported social skills.

The pallidum, part of the basal ganglia, is involved in regulating voluntary and proprioceptive movements and plays a role in motor, cognitive, and reward circuits (23). Its dysfunction is linked to various neurological and psychiatric disorders like ischemia, alcohol and opiate abuse, obsessive-compulsive disorder (OCD), Tourette’s syndrome, acquired dystonia, and attention deficit hyperactivity disorder (ADHD). It also has implications for the motor symptoms of Parkinson’s disease.

In MS, Motl et al. found that pallidum volume strongly correlated with walking performance, suggesting its involvement in motor function (24). Moreover, Krutenkova et al. reported reduced myelination in the pallidum in MS patients compared to healthy controls (25). Interestingly, Fleischer et al. identified pallidal volume as a predictor of fatigue severity after the first demyelinating event (26).

On the other hand, in our study, thalamic atrophy was observed in patients with EDSS progression. Moreover, when analyzing the three-year changes in volume with changes in the EDSS scale, a greater atrophy of the accumbens area was observed in patients with disease progression measured by EDSS. Furthermore, taking into account the first two years of follow-up, atrophy of the subcortical GM was observed in patients with an increase in EDSS. Remarkably, in the CLAD group, a significant negative correlation was observed between EDSS changes and a change in the volume of the cerebellar cortex, which was not observed in the ALEM group, where it correlated with thalamus atrophy.

These results aligned with Cagol et al., who found that EDSS changes correlated most with deep GM, especially thalamic volume, even in PIRA patients (3). This was confirmed by another work by this author, in which, compared to healthy controls, MS patients showed significant thalamic changes, indicating microstructural and macrostructural damage, demyelination and disruption of iron homeostasis (27). The greatest thalamic degeneration was observed in patients with PPMS. Moreover, the progression of disability during the follow-up period was associated with accelerated thalamic degeneration. Eshaghi et al. confirmed thalamic atrophy as the strongest predictor of EDSS progression across all MS subtypes, making it a key marker of worsening disability (28). This was consistent with previous results by these authors, where patients with MS showed a greater decrease in GM volume (p < 0.05, FWE corrected) including in the thalamus compared to healthy controls over the five-year follow-up (29). Similarly, Rocca and Hanninen demonstrated that baseline thalamic volume predicted long-term disability (30, 31). This suggested that thalamic atrophy might correlate with long-term progression of disability in MS patients. Furthermore, other studies (e.g., Schoonheim et al.) have linked lower thalamic volume with cognitive impairment (32–34). In addition, Niiranen et al. identified thalamic volume as key in distinguishing mild from aggressive RRMS (35). In PPMS, thalamic atrophy was also a common early marker, as shown by Khaleeli et al. and Sepulcre et al. (36, 37). Interestingly, Ramezani et al. demonstrated an association between the normalized volume of the whole thalamus and MS subtype (38). Furthermore, the results of their study suggested that thalamic asymmetry may be associated with disease progression and subtype changes in MS.

Overall, thalamic volume reflects both disease severity and clinical heterogeneity across MS types and is involved in the earliest form of the disease, radiologically isolated syndrome (RIS) (39). It correlates with fatigue, movement disorders, cognitive deficits, and may result from secondary degeneration due to white matter damage. It also may represent a promising biomarker for neurodegeneration and treatment monitoring, supported by studies like Nakamura et al., who found reduced thalamic volume loss under natalizumab therapy (40).

The nucleus accumbens, part of the ventral striatum between the putamen and caudate nucleus, plays a central role in the brain’s reward system, motivation, and emotional regulation (41). It links the limbic and motor systems and is involved in functions such as pleasure, addiction, impulsivity, and survival behaviors. It is also implicated in psychiatric conditions like schizophrenia, depression, and Parkinson’s disease, and has become a target for some psychosurgical treatments.

Lorefice et al. found reduced accumbens volume, especially in MS patients with bipolar symptoms, suggesting a link between DGM atrophy and psychiatric comorbidities (42). Furthermore, Seixas et al. described functional and structural changes in the reward circuit, including the accumbens, in MS patients with chronic pain (43).

Remarkably, in the presented study, 10 patient-years with EDSS progression were identified between time points 1-3. Among them, 3 patients showed progression not related to disease activity (PIRA). The remaining 7 patients experienced disease progression measured by EDSS and associated with RAW. An increase in amygdala volume was observed in all three patients with PIRA, whereas this phenomenon was not reported in the seven patients whose EDSS progression was associated with recurrent activity. The study by Cagol et al. (2022) showed that patients with RMS and PIRA exhibit accelerated brain atrophy, particularly in the cerebral cortex (3). Patients with PIRA also showed more rapid ventricular enlargement compared to stable patients. In addition, accelerated thinning was detected throughout the cortex, as well as in temporal, frontal, parietal, insular, and cingulate cortices. In the subgroup of patients with PIRA who had no radiological inflammatory activity throughout the follow-up period, investigators continued to observe accelerated rates of atrophy compared with the matched group of stable patients. These results indicated the need to recognize the insidious manifestations of PIRA in clinical practice and to further evaluate treatment strategies for patients with PIRA in clinical trials. In another study, Cagol (2023) confirmed that accelerated brain atrophy was detected in patients experiencing PIRA (44). PIRA was associated with both increased spinal cord atrophy and paramagnetic rim lesions (PRLs) burden. These results further underscore the need to develop targeted treatment strategies for PIRA to prevent irreversible neuroaxonal loss and optimize long-term outcomes in patients with MS.

The present study had several limitations. First, measures of spinal cord atrophy and cortical changes were not included, although they have been previously shown to predict physical disability and disease progression, asymptomatic progression, and conversion to SPMS (41). Furthermore, the criterion used to define PIRA was based only on the EDSS score. Given the lack of upper and lower limb function measures in our cohort, subtle neurological deterioration that did not result in an increase in EDSS score may have been missed. Another aspect was the relatively small size of the analyzed group. This was due to the fact that it was a retrospective study based on real-world evidence (RWE). In addition, some data from the analysis had to be excluded to control for potential confounding factors that could distort the assessment of the pattern and degree of atrophy in the compared reconstitution therapies. For this reason, MRI examinations performed less than 8 weeks after the administration of intravenous steroids were not included in the data analysis to reduce the phenomenon of pseudoatrophy. MRI examinations of poor technical quality and performed at incorrect intervals were also excluded to minimize the risk of artifacts that could affect the obtained results. Although all limitations reduced the size of the analyzed group, they increased the reliability of the results. The presented study can therefore be considered a pilot study in which no specific hypotheses were assumed in advance, but it was an exploratory analysis. Therefore, further prospective studies with larger patient groups are needed.

The presented study seems to be innovative due to the finding of a strong correlation between the change in the volume of the amygdala (and to a lesser extent the pallidum) and changes in EDSS. However, as already highlighted above, this was an exploratory study on a small number of patients and its results provide only certain hypotheses. Currently, there is a lack of data in the literature assessing this type of correlation. In the discussion, we presented single studies describing the relationship between these structures and the assessment of disease progression (13, 24). In addition, this research establishes the role of subcortical structures such as the DGP or thalamus as markers that can predict the progression of disability. By eliminating confounding factors, we tried to present the most reliable data. To our knowledge, our analysis is one of the few national RWE studies trying to find connections between volumetric changes in brain areas and clinical dynamics in patients with MS. In summary, presented results confirm thalamic atrophy and DGM as important parameters predicting disability progression in MS. They also shed new light on the possible potential relationship between changes in the volume of structures such as the amygdala with the progression of disability measured by the EDSS scale and the occurrence of the PIRA phenomenon. However, further research are needed to fully understand these correlations and their possible use in monitoring the course of the disease as well as the response to treatment. In addition, while our study was not designed to assess the prognostic utility of baseline MRI features, our findings raised the possibility that early volumetric markers—particularly amygdala volume—may help identify RRMS patients at risk of disease progression. Therefore, future studies should investigate whether baseline morphology, texture, or connectivity patterns of individual areas of the brain can predict subsequent volume changes and disability progression. Machine learning approaches, such as regularized regression or survival models incorporating baseline volumetric and clinical variables, could be employed to classify patients into risk subgroups. If validated, such models may ultimately support early, individualized treatment decisions and stratification in clinical trials.

## Data Availability

The original contributions presented in the study are included in the article/**Supplementary Material**. Further inquiries can be directed to the corresponding author.
