# Supplementary material for: Amygdala volume changes as a potential marker of multiple sclerosis progression: links to EDSS scores and PIRA
Source: Front Immunol. 2025 Aug 26;16:1640607. doi: 10.3389/fimmu.2025.1640607 (PMC12417161; doi:10.3389/fimmu.2025.1640607)
Supplement: Supplementary file 1 [file Table1.docx]

Supplementary Material

|  | **ALL** | | | **CLAD** | | | **ALEM** | | |
| --- | --- | --- | --- | --- | --- | --- | --- | --- | --- |
|  | **η²** | **p_value** | **n** | **η²** | **p_value** | **n** | **η²** | **p_value** | **n** |
| **Accumbens** | 0,00222 | 0,65559 | 97 | 0,00002 | 0,97580 | 60 | 0,00795 | 0,62163 | 37 |
| **Amygdala** | 0,15657 | 0,00009 | 97 | 0,05963 | 0,06972 | 60 | 0,24923 | 0,00310 | 37 |
| **Pallidum** | 0,05384 | 0,02605 | 97 | 0,05413 | 0,08444 | 60 | 0,06511 | 0,15180 | 37 |
| **Cerebellar WM** | 0,02681 | 0,11885 | 97 | 0,06295 | 0,06217 | 60 | 0,00496 | 0,69702 | 37 |
| **Thalamus** | 0,02326 | 0,14665 | 97 | 0,07203 | 0,04551 | 60 | 0,00371 | 0,73616 | 37 |
| **Putamen** | 0,01765 | 0,20680 | 97 | 0,02659 | 0,22980 | 60 | 0,02001 | 0,43235 | 37 |
| **CC** | 0,01051 | 0,33081 | 97 | 0,00784 | 0,51651 | 60 | 0,03162 | 0,32213 | 37 |
| **VentralDC** | 0,00562 | 0,47722 | 97 | 0,02117 | 0,28463 | 60 | 0,00213 | 0,79883 | 37 |
| **CSF** | 0,00089 | 0,77800 | 97 | 0,00460 | 0,61950 | 60 | 0,00001 | 0,98697 | 37 |
| **Cortex** | 0,00050 | 0,83296 | 97 | 0,00011 | 0,93958 | 60 | 0,00004 | 0,97189 | 37 |
| **Total GM** | 1,59727 | 0,99046 | 97 | 8,24809 | 0,98324 | 60 | 0,00160 | 0,82523 | 37 |
| **DGM** | 0,00352 | 0,57433 | 97 | 0,06859 | 0,05120 | 60 | 0,11350 | 0,05522 | 37 |
| **Caudate** | 0,01297 | 0,27965 | 97 | 0,02688 | 0,22728 | 60 | 0,10269 | 0,06904 | 37 |
| **Cerebral WM** | 0,00182 | 0,68606 | 97 | 0,00047 | 0,87424 | 60 | 0,00586 | 0,67190 | 37 |
| **Total WM** | 0,00118 | 0,74537 | 97 | 0,00010 | 0,94142 | 60 | 0,00500 | 0,69579 | 37 |
| **Cerebrum** | 0,00408 | 0,54534 | 97 | 0,00083 | 0,83326 | 60 | 0,02289 | 0,40068 | 37 |
| **Hippocampus** | 0,00683 | 0,43340 | 97 | 0,05590 | 0,07937 | 60 | 0,08110 | 0,10819 | 37 |
| **Cerebellum** | 0,00027 | 0,87599 | 97 | 0,00810 | 0,50938 | 60 | 0,02633 | 0,36694 | 37 |

**Supplementary Table S1. Year-to-year correlations between Expanded Disability Status Scale (EDSS) changes and volume changes.**P-value and eta-squared (η²) were obtained from a linear model and are adjusted by sex, age, baseline EDSS, and number of previous disease modifying therapies (DMTs).
Cerebellar WM - volume of cerebellar white matter, CC- corpus callosum, Ventral DC - ventral diencephalon, CSF - cerebrospinal fluid, Total GM - total volume of gray matter, DGM - volume of subcortical gray matter, Cerebral WM – volume of white matter of brain, Total WM – total white matter volume, η² - eta- squared

|  | **p_value** | **η²** | **levene** |
| --- | --- | --- | --- |
| **Accumbens** | 0,54995 | 0,01335 | 0,24379 |
| **Amygdala** | 0,00640 | 0,10733 | 0,64920 |
| **Pallidum** | 0,02904 | 0,07645 | 0,38506 |
| **Cerebellar WM** | 0,43801 | 0,01838 | 0,79384 |
| **Thalamus** | 0,04390 | 0,06783 | 0,09508 |
| **Putamen** | 0,32098 | 0,02521 | 0,10031 |
| **CC** | 0,08925 | 0,05285 | 0,06377 |
| **VentralDC** | 0,74349 | 0,00664 | 0,27336 |
| **CSF** | 0,96608 | 0,00076 | 0,66417 |
| **Cortex** | 0,92504 | 0,00175 | 0,54318 |
| **Total GM** | 0,83954 | 0,00392 | 0,34426 |
| **DGM** | 0,60995 | 0,01105 | 0,22322 |
| **Caudate** | 0,07896 | 0,05546 | 0,16519 |
| **Cerebral WM** | 0,60995 | 0,01105 | 0,22322 |
| **Total WM** | 0,92618 | 0,00172 | 0,48398 |
| **Cerebrum** | 0,90956 | 0,00213 | 0,21654 |
| **Hippocampus** | 0,35255 | 0,02316 | 0,04545 |
| **Cerebellum** | 0,78143 | 0,00553 | 0,24742 |

**Supplementary Table S2. Comparison of annual percentage volume changes in the amygdala, thalamus, and pallidum, grouped by annual Expanded Disability Status Scale (EDSS) change: regression(n = 10), progression (n = 11), and stabilization (n = 74).**The division into groups with regression or progression of EDSS was defined as for PIRA. P-values and effect sizes (eta-squared (η²)) were obtained from an ANCOVA-like linear model and are adjusted by sex, age, baseline EDSS, and number of previous disease modifying therapies (DMTs). The final column reports Levene’s test for equality of variances. Cerebellar WM - volume of cerebellar white matter, CC- corpus callosum, Ventral DC - ventral diencephalon, CSF - cerebrospinal fluid, Total GM - total volume of gray matter, DGM - volume of subcortical gray matter, Cerebral WM – volume of white matter of brain, Total WM – total white matter volume, η² - eta- squared

|  | **ALL** | | | **CLAD** | | | **ALEM** | | |
| --- | --- | --- | --- | --- | --- | --- | --- | --- | --- |
|  | **slope** | **p_value** | **n** | **slope** | **p_value** | **n** | **slope** | **p_value** | **n** |
| **Accumbens** | -0.004496 | 0.004729 | 16 | -0.003546 | 0.183391 | 9 | -0.004344 | 0.164171 | 7 |
| **Amygdala** | 0.007408 | 0.100005 | 16 | 0.004935 | 0.446728 | 9 | -0.001331 | 0.867344 | 7 |
| **Pallidum** | 0.014928 | 0.167143 | 16 | 0.023624 | 0.139916 | 9 | 0.013639 | 0.595771 | 7 |
| **Cerebellar WM** | -0.058299 | 0.125633 | 16 | -0.160321 | 0.016256 | 9 | -0.063693 | 0.335631 | 7 |
| **Thalamus** | -0.013744 | 0.536467 | 16 | -0.043677 | 0.214945 | 9 | 0.002264 | 0.964312 | 7 |
| **Putamen** | -0.011155 | 0.363053 | 16 | -0.034699 | 0.099525 | 9 | -0.006033 | 0.811994 | 7 |
| **CC** | -0.009781 | 0.553158 | 16 | -0.015668 | 0.540845 | 9 | -0.045309 | 0.165980 | 7 |
| **VentralDC** | 0.000305 | 0.948883 | 16 | -0.001881 | 0.854199 | 9 | -0.001860 | 0.806616 | 7 |
| **CSF** | 0.002572 | 0.662606 | 16 | 0.001493 | 0.843625 | 9 | -0.010955 | 0.373611 | 7 |
| **Cortex** | -0.855879 | 0.179017 | 16 | -0.842764 | 0.279751 | 9 | 0.633971 | 0.619418 | 7 |
| **Total GM** | -0.761140 | 0.247719 | 16 | -0.728934 | 0.426676 | 9 | 0.721831 | 0.570328 | 7 |
| **DGM** | 0.016306 | 0.668102 | 16 | -0.051790 | 0.347340 | 9 | 0.002519 | 0.975433 | 7 |
| **Caudate** | 0.011170 | 0.164590 | 16 | -0.001609 | 0.921645 | 9 | 0.025978 | 0.040177 | 7 |
| **Cerebral WM** | 1.251780 | 0.193973 | 16 | 1.088100 | 0.356498 | 9 | -0.367101 | 0.864465 | 7 |
| **Total WM** | 1.193481 | 0.223531 | 16 | 0.927779 | 0.439702 | 9 | -0.430793 | 0.843745 | 7 |
| **Cerebrum** | 0.501961 | 0.296687 | 16 | 0.193800 | 0.842137 | 9 | 0.467369 | 0.579103 | 7 |
| **Hippocampus** | 0.006905 | 0.454676 | 16 | 0.002023 | 0.698946 | 9 | -0.021714 | 0.219378 | 7 |
| **Cerebellum** | 0.025326 | 0.845889 | 16 | -0.063687 | 0.798938 | 9 | 0.043806 | 0.864135 | 7 |

**Supplementary Table S3.** Correlations between three-year changes in volume and changes in the Expanded Disability Status Scale (EDSS). Cerebellar WM - volume of cerebellar white matter, CC- corpus callosum, Ventral DC - ventral diencephalon, CSF - cerebrospinal fluid, Total GM - total volume of gray matter, DGM - volume of subcortical gray matter, Cerebral WM – volume of white matter of brain, Total WM – total white matter volume

|  | **ALL** | | | **CLAD** | | | **ALEM** | | |
| --- | --- | --- | --- | --- | --- | --- | --- | --- | --- |
|  | **slope** | **p_value** | **n** | **Slope** | **p_value** | **n** | **slope** | **p_value** | **n** |
| **Accumbens** | -0.002466 | 0.046096 | 28 | -0.001029 | 0.421473 | 18 | -0.003473 | 0.120290 | 10 |
| **Amygdala** | 0.005465 | 0.090801 | 28 | 0.004602 | 0.168752 | 18 | 0.005525 | 0.391266 | 10 |
| **Pallidum** | 0.012103 | 0.132616 | 28 | 0.007902 | 0.204525 | 18 | 0.017502 | 0.346771 | 10 |
| **Cerebellar WM** | -0.036641 | 0.380991 | 28 | 0.019432 | 0.748592 | 18 | -0.092244 | 0.141272 | 10 |
| **Thalamus** | -0.042300 | 0.001176 | 28 | -0.024319 | 0.108546 | 18 | -0.061922 | 0.011247 | 10 |
| **Putamen** | -0.014940 | 0.070088 | 28 | -0.011484 | 0.153036 | 18 | -0.019177 | 0.286711 | 10 |
| **CC** | -0.004993 | 0.640091 | 28 | 0.000116 | 0.992915 | 18 | -0.011209 | 0.578975 | 10 |
| **VentralDC** | -0.004301 | 0.330427 | 28 | -0.004213 | 0.423155 | 18 | -0.004070 | 0.638763 | 10 |
| **CSF** | -0.006114 | 0.122665 | 28 | 0.002865 | 0.499203 | 18 | -0.014801 | 0.051152 | 10 |
| **Cortex** | -0.235637 | 0.642249 | 28 | -0.001838 | 0.997374 | 18 | -0.288283 | 0.746902 | 10 |
| **Total GM** | -0.366282 | 0.472723 | 28 | -0.201192 | 0.726369 | 18 | -0.339244 | 0.692870 | 10 |
| **DGM** | -0.051146 | 0.000779 | 28 | -0.050201 | 0.010769 | 18 | -0.056734 | 0.019775 | 10 |
| **Caudate** | -0.004661 | 0.513289 | 28 | -0.019162 | 0.048056 | 18 | 0.008805 | 0.418455 | 10 |
| **Cerebral WM** | -0.125400 | 0.871434 | 28 | -0.290134 | 0.778805 | 18 | -0.157075 | 0.899350 | 10 |
| **Total WM** | -0.162041 | 0.837789 | 28 | -0.270702 | 0.795900 | 18 | -0.249320 | 0.846743 | 10 |
| **Cerebrum** | -0.331412 | 0.371918 | 28 | -0.391761 | 0.509458 | 18 | -0.268982 | 0.561471 | 10 |
| **Hippocampus** | -0.000274 | 0.954762 | 28 | 0.000146 | 0.979445 | 18 | -0.002600 | 0.732540 | 10 |
| **Cerebellum** | -0.108668 | 0.128709 | 28 | -0.142391 | 0.149708 | 18 | -0.061487 | 0.584041 | 10 |

**Supplementary Table S4.** Correlations between volume and Expanded Disability Status Scale (EDSS) changes in the first two years of observation. Cerebellar WM - volume of cerebellar white matter, CC- corpus callosum, Ventral DC - ventral diencephalon, CSF - cerebrospinal fluid, Total GM - total volume of gray matter, DGM - volume of subcortical gray matter, Cerebral WM – volume of white matter of brain, Total WM – total white matter volume

|  | **Sample size** | **η²** |
| --- | --- | --- |
| **Accumbens** | 238 | 0.0133 |
| **Amygdala** | 28 | 0.107 |
| **Pallidum** | 40 | 0.076 |
| **Cerebellar WM** | 173 | 0.018 |
| **Thalamus** | 45 | 0.068 |
| **Putamen** | 125 | 0.025 |
| **CC** | 59 | 0.053 |
| **VentralDC** | 482 | 0.007 |
| **CSF** | 4141 | 0.001 |
| **Cortex** | 1833 | 0.002 |
| **Total GM** | 817 | 0.004 |
| **DGM** | 288 | 0.011 |
| **Caudate** | 55 | 0.055 |
| **Cerebral WM** | 2301 | 0.001 |
| **Total WM** | 1863 | 0.001 |
| **Cerebrum** | 1507 | 0.002 |
| **Hippocampus** | 136 | 0.023 |
| **Cerebellum** | 579 | 0.006 |

**Supplementary Table S5. Estimated number of study participants per group necessary to detect statistically significant differences, assuming the effect sizes observed in our study, using one-way analysis of variance with α = 0.05 and a power of 80%.**Cerebellar WM - volume of cerebellar white matter, CC- corpus callosum, Ventral DC - ventral diencephalon, CSF - cerebrospinal fluid, Total GM - total volume of gray matter, DGM - volume of subcortical gray matter, Cerebral WM – volume of white matter of brain, Total WM – total white matter volume, η² - eta- squared
